# Assessing school preparedness for disaster resilience: Integrating Gender, Disability, and Social Inclusion (GEDSI) in the safe school programme

**DOI:** 10.4102/jamba.v18i1.1927

**Published:** 2026-06-10

**Authors:** Muhammad Musiyam, Siti H.N. Hafida, Siti Aisyah

**Affiliations:** 1 Department of Geography Education, Faculty of Teacher Training and Education, Universitas Muhammadiyah Surakarta, Sukoharjo, Indonesia; 2 Department of Disaster Mitigation Study Center, Universitas Muhammadiyah Surakarta, Sukoharjo, Indonesia; 3 Department of Economics, Faculty of Economics and Business, Universitas Muhammadiyah Surakarta, Sukoharjo, Indonesia

**Keywords:** gender, disability, social inclusion, safe schools programme, Indonesia

## Abstract

Schools play a critical role in disaster risk reduction; however, preparedness efforts often overlook the unequal vulnerabilities associated with Gender, Disability, and Social Inclusion. This study addresses the lack of empirical evidence on how inclusively disaster preparedness is implemented within school-based disaster management structures, particularly the Safe School Disaster Unit (SSDU). Using a quantitative survey design, data were collected from senior high schools to assess levels of SSDU readiness, the integration of Gender, Disability, and Social Inclusion (GEDSI) principles and the relationship between these two dimensions. This study used a quantitative design. Data were collected through questionnaires from 995 respondents across five schools and analysed using regression analysis. The findings reveal substantial variation in preparedness across schools, with public schools generally demonstrating higher levels of SSDU readiness and GEDSI integration than private schools. Statistical analysis further indicates a positive association between stronger SSDU implementation and higher levels of GEDSI integration, suggesting that schools with more established disaster management systems are better positioned to adopt inclusive practices. These results highlight persistent gaps in inclusive disaster preparedness and underscore the need to strengthen institutional systems and policies that embed GEDSI principles within school disaster risk reduction initiatives. The study contributes empirical evidence to inform more equitable and resilient safe school programming.

**Contribution:**

Disaster risk reduction strategies in school environments have so far focused on physical and structural aspects, whereas, on the other hand, there are aspects that have not been studied, such as Gender, Disability, and Social Inclusion. Therefore, it is important to know the implementation of safe schools by focusing on aspects of Gender, Disability, and Social Inclusion in order to realise school resilience to disasters in a fair, equal and sustainable manner.

## Introduction

Children’s vulnerability in multiple settings, especially in schools, has been significantly amplified by the increasing frequency and intensity of disasters. Schools are vital institutions that play an important role in protecting children during disasters (Hoffmann & Blecha 2020; Seddighi et al. 2021). However, in the context of disasters, schools are often vulnerable. Natural hazards such as earthquakes, floods and hurricanes can affect the physical safety of schools as well as the psychological well-being of students (Bhebhe, Runhare & Monobe 2019; Hafida, Isa & Khotimah 2022; Hassan, Singh & Sekar 2018; Maynard et al. 2019; Wijayanti & Hafida 2023). In fact, according to data from the Indonesian Disaster Data Geoportal (Geoportal Data Bencana Indonesia 2024), natural hazards that have occurred in Indonesia have impacted 423 educational units. Some of the disasters that have occurred in Indonesia and have impacted the sustainability of schools include: In 2006, the Yogyakarta Earthquake affected the education sector by 2155 students. In addition, during the eruption of Mount Merapi, it was discovered that schools on the slopes of Mount Merapi were closed for almost 2 weeks because the schools were used as shelters for disaster-affected communities (United Nations Office for Disaster Risk Reduction [UNDRR] 2020).

Given that children spend most of their time in school (Dania et al. 2022; Pulimeno et al. 2020; Shah et al. 2018), school preparedness in facing disasters becomes a crucial issue. This issue ultimately encourages schools to be able to provide safe and comfortable learning for students. Most developed countries, such as Japan and the United States, use education to prepare children to face natural hazards (Gavari-Starkie, Casado-Claro & Navarro-González 2021; Sakurai 2016). Education is very important in preparing for and managing disasters (Bhebhe et al. 2019). Education is used to convey knowledge to students, and the education sector also has an important role in educating and building cooperation between students in disaster emergencies (Mirzaei et al. 2019). Therefore, there is a Safe School Disaster Unit (SSDU) programme to minimise disasters in the educational environment by providing appropriate guidance and mitigation strategies (Global Alliance for Disaster Risk Reduction and Resilience in the Education Sector [GADDRESS] 2022). The government initiated this programme to ensure that each educational unit can manage disaster risks, protect students and staff and minimise the impact of disasters (Amri et al. 2022).

Safe School Disaster Unit itself has three pillars, namely Pillar 1 (safer learning facilities), Pillar 2 (school safety and educational continuity management) and Pillar 3 (risk reduction and resilience education) (GADDRESS 2022; Koswara et al. 2019). Safe School Disaster Unit aims to protect and improve students’ knowledge and skills to contribute to disaster risk reduction (DRR), disaster resilience development and sustainable development (GADDRESS 2022; Gavari-Starkie et al. 2021). Although the SSDU programme has been implemented in many schools, the level of school readiness in implementing it still varies. Some schools have sufficient infrastructure, while others need more resources such as facilities and knowledge about disaster mitigation. School readiness also includes components of disaster risk management, teacher capacity and school community participation in responding to disaster threats (Amri et al. 2020, 2022). This shows the need to assess school readiness to ensure that each institution can protect students from disaster risks.

Schools that can create a sense of security for their students can be an effective effort to reduce disaster risks (Hermon et al. 2019). An important aspect often overlooked in implementing SSDU is Gender Equality, Disability, and Social Inclusion (GEDSI). Each student has different vulnerabilities based on their social identity, including gender, disability or marginalised groups. Female students, children with disabilities and underserved groups often face greater barriers in emergencies (Azmi 2023; Innovation for Indonesia’s School Children [INOVASI] 2020). Therefore, integrating GEDSI into SSDU policies is essential to ensure that all students, without exception, are protected and have access to the support they need when a disaster occurs. Active involvement of the entire school community can reduce 40% – 60% of disaster losses (Catalano, Forni & Pezzolla 2020; Pilli-Sihvola, Harjanne & Haavisto 2018).

Gender inequality, disability and social marginalisation can heighten vulnerability during disasters by shaping unequal access to resources, participation and protection within school settings. Gender norms may limit the participation of girls in disaster preparedness training or decision-making processes, while students with disabilities often face physical barriers, limited access to early warning information and inadequate evacuation support. Similarly, socially marginalised groups may experience exclusion from communication channels and school-based safety planning. When these factors intersect, they can delay response actions, restrict mobility and reduce the ability of vulnerable students to access timely assistance during emergencies, thereby exacerbating disaster impacts within schools.

In emergencies, women are often at greater risk of violence or exclusion (Cvetković et al. 2018), requiring special attention in terms of safety, health and well-being during and after disasters (Bali 2021; Hafida 2019). While children with disabilities may not be able to access appropriate evacuation routes (Jang & Ha 2021), people with disabilities face extra barriers in accessing information, transport, shelter and health services during disasters. Furthermore, a lack of awareness and understanding of these differential vulnerabilities among school staff can exacerbate the situation. Even the participation of women and marginalised groups in national, provincial and local DRR structures is very low (Chet et al. 2023; Hafida 2019; Susilongtyas et al. 2020). This low involvement is because of a lack of understanding of the root causes of vulnerability (UN Women 2022). Without a GEDSI strategy, the risk of vulnerability is higher for these groups, thereby compromising the overall disaster resilience of schools (Crawford et al. 2023).

Integrating GEDSI into school DRR policies can significantly improve disaster preparedness and resilience. Through an inclusion-based approach, schools can ensure that the needs of every student, including those who are vulnerable, are addressed equitably. By integrating GEDSI strategies into SSDU, not only girls, marginalised groups and people with disabilities are better protected but also the entire school community is more responsive to disaster risks, strengthens social cohesion and minimises the negative impacts of disasters. This article aims to assess school disaster preparedness through the SSDU framework, with a particular focus on the integration of GEDSI aspects. This study will highlight how schools have implemented gender friendly, inclusive policies and procedures that can protect students with disabilities in facing disasters. With an in-depth analysis of school preparedness in the context of GEDSI, this article is expected to enrich the literature on disaster-safe education and provide more inclusively practical recommendations for policymakers and schools in implementing SSDU programmes.

### Gender, Disability, and Social Inclusion in Indonesia

The Sendai Framework for Disaster Risk Reduction (SFDRR) 2015–2030 is the main guideline for DRR. The SFDRR explicitly emphasises the importance of a gender-sensitive, disability-sensitive and social inclusion approach in all stages of disaster management. This framework is also in line with the 2030 Agenda for Sustainable Development Goals (SDGs), especially SDG 5 (gender equality), SDG 10 (reduced inequalities) and SDG 13 (action on climate change). Gender, Disability, and Social Inclusion (GEDSI) is a concept that integrates gender equality, the rights of persons with disabilities and social inclusion in various aspects of development. Several policies that have been integrated with the GEDSI approach in Indonesia include: (1) *Law Number 24 of 2007* concerning disaster management, (2) the National Disaster Management Plan 2020–2024, (3) *Law Number 19 of 2011* concerning the Ratification of the UN Convention on the Rights of Persons with Disabilities (CRPD), (4) *Law Number 8 of 2016* concerning Persons with Disabilities, (5) *Presidential Regulation Number 87 of 2020* concerning the Master Plan for Disaster Management 2020–2044, (6) *Government Regulation Number 42 of 2020* and (7) Regulation of the Head of the National Disaster Management Agency Number 14 of 2014.

Gender, Disability, and Social Inclusion is an important approach to creating an inclusive and disaster-resilient education system in Indonesia. Gender, Disability, and Social Inclusion can promote equity and inclusion in education and mitigate disaster risks for vulnerable groups. Joint commitment from all parties is needed to ensure the successful implementation of GEDSI, so that a more just and inclusive society will be created. Based on existing literature, there are six main variables of GEDSI, including: (1) *School commitment*, inclusive schools that are committed to implementing GEDSI conduct training for teachers and staff to understand the needs of students with special needs and promote a culture of gender equality (Roy & Mukherjee 2024), (2) *Curriculum*, GEDSI-based curriculum integrates values of inclusivity, such as tolerance, respect for diversity and the importance of active participation of all students, including those with disabilities. The education curriculum should include emergency response training, such as inclusive evacuation simulations for students with physical disabilities (Crawford et al. 2023), (3) *Information dissemination*, dissemination of disability-friendly information, such as using audio-visual media and sign language, helps to create an inclusive learning environment (Cui et al. 2020; Hwang et al. 2018), (4) *School facilities*, the construction of inclusive school facilities, such as wheelchair ramps, special toilets and sensory-friendly learning spaces, are concrete steps to support GEDSI (Henderson & Barnes 2015), (5) *Monitoring system*, monitoring of GEDSI implementation is carried out by educational institutions and related parties to ensure that policies are implemented effectively and evenly (Rashid & Shafie 2013; Zaidi & Fordham 2021) and (6) *Collaboration*, collaboration between the government, community organisations and donor agencies encourages inclusive education, including in the provision of resources and training (Henderson & Barnes 2015; Jang & Ha 2021).

### School safety framework in Indonesia

In Indonesia, the School Safety Framework is an important component in ensuring that schools are well-prepared for disaster resilience, particularly within the framework of the SSDU programme. This framework is aligned with the Comprehensive School Safety Framework (CSSF) initiated by the GADDRESS. It has been adopted globally to guide DRR in education. In Indonesia, this programme is important given Indonesia’s high level of vulnerability to various types of disasters, such as earthquakes, floods, volcanic eruptions, landslides, tsunamis and other disasters. The main elements of the SSDU assessment in Indonesia include three pillars, including:

#### Safer school facilities

This pillar focuses on ensuring that school infrastructure is resilient to natural hazards. This pillar will focus on the condition of the building and all its contents that meet the criteria for building reliability by existing policies (GADDRESS 2022; Tebe et al. 2023). In Indonesia, frequent disasters such as earthquakes, floods, landslides and volcanic eruptions have highlighted the need for strong building codes and the remediation of vulnerable school buildings. Buildings that are easily damaged will certainly increase the vulnerability of the existing school community (Chet et al. 2023; Dewa et al. 2023). The Ministry of Education and the National Disaster Management Agency (BNPB) have attempted to assess the structural integrity of school buildings, especially in disaster-prone areas. The purpose of this pillar is to minimise the risk of injury or loss of life because of structural and non-structural damage (Nakum et al. 2022; Paci-Green et al. 2020; Tong et al. 2024).

#### Disaster management and educational continuity

This aspect discusses equality for learners’ safety, health and well-being in educational continuity. This pillar focuses on developing anticipatory, absorptive, adaptive and transformative capacities for resilience (GADDRESS 2022). This pillar involves the creation of disaster preparedness and response plans, training for teachers and students and the implementation of regular disaster simulations (Paci-Green et al. 2020). In addition, this pillar also includes activities to conduct disaster risk assessments and disaster recovery and act quickly and efficiently (Tong et al. 2024). Schools that implement this pillar can help improve positive attitudes in students (Chet et al. 2023).

#### Risk reduction and resilience education

This pillar focuses on efforts to create content, processes and learning opportunities for children to support resilience according to the risks they face. According to GADDRESS (2022), this pillar is considered part of the thinking and practical efforts to eliminate or reduce all risks by prioritising the learning process or other educational activities so that a culture of preparedness is realised in facing various threats of danger from a disaster. This pillar emphasises the provision of knowledge that will later influence the attitudes and skills of students, teachers and even parents to create a culture of safety (Nakum et al. 2022; Tong et al. 2024). This involves integrating disaster risk education into the curriculum, emphasising awareness and preparedness. In Indonesia, this is a growing focus, and students are taught about local hazards and the importance of inclusivity in responding to disasters.

## Research methods and design

This study employs a quantitative survey design to assess school preparedness for disaster resilience and the implementation of GEDSI within the SSDU. A quantitative approach is appropriate as it enables systematic measurement of multiple preparedness and inclusivity dimensions using standardised indicators, allowing for objective comparison across schools. Compared to qualitative methods, this approach provides clearer insights into overall levels, patterns and variations of SSDU readiness and GEDSI implementation. It also facilitates statistical analysis of the relationship between disaster preparedness structures and inclusive practices, thereby strengthening the empirical basis of the findings. Furthermore, the quantitative design enhances the replicability and generalisability of results, which are essential for informing policy and programmatic decision-making. This is in accordance with the statement of Llorente-Marrón et al. (2020) that quantitative analysis is very important in disaster research because it can provide empirical evidence to understand the level of inequality effectively. This method allows statistical data analysis, making it easier to understand the trend of inequality. The respondents in this study were 995 students who were selected proportionally from five high schools located in disaster-prone areas. The sample selection was conducted using proportional sampling, so that each school contributed a sample proportionate to its population size. The GEDSI-based school readiness measurement model requires a large sample size to more easily generalise findings on variations in conditions between schools in disaster-prone areas.

### Research location

**FIGURE 1:**
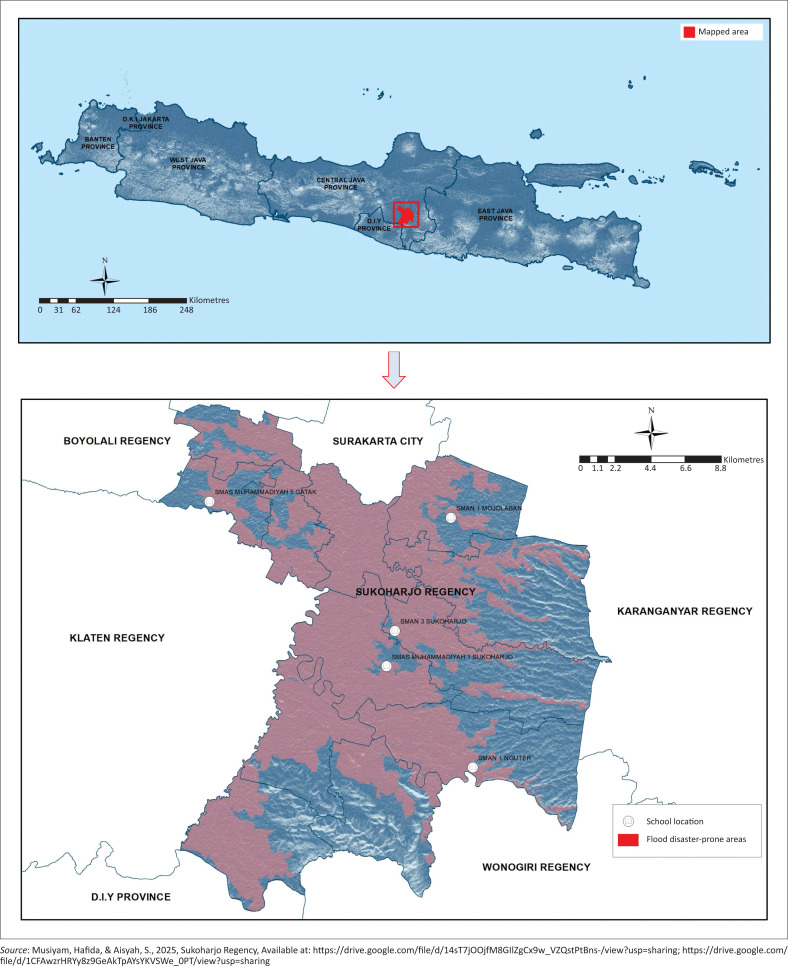
Research location map.

This study was conducted in five schools in Sukoharjo Regency, namely Public Senior High School 1 Mojolaban, Private Senior High School 5 Gatak, Public Senior High School 1 Nguter, Private Senior High School 1 Sukoharjo and Public Senior High School 3 Sukoharjo (Figure 1). The selection of the research location was adjusted to the existing disaster risk index so that the five school locations were obtained. In addition, the school’s location is in the southern part of Java, where, according to Musiyam (2020), the southern part of Java is highly vulnerable to disasters.

### Data collection instruments

Before collecting data, the researcher conducted a pilot study to ensure that the instrument used was appropriate. This pilot study was conducted to check the validity and reliability of the questionnaire (Raphela 2024). The pilot study was conducted at SMA Negeri 1 Mojolaban with 54 students as respondents. The results of the pilot study showed that the questionnaire was good and could be used as a data collection instrument. In addition, the researcher also ensured integration with policies such as safe schools by the GADDRESS (2022).

*Questionnaires* were used to measure behaviour, preferences and facts (Taherdoost 2021). The questionnaire was given to students and used to determine student perceptions regarding implementing SSDU and managing GEDSI in schools. *Interviews* were conducted by asking questions about respondents’ experiences, opinions and expectations regarding a particular problem (Knott et al. 2022). Interviews were conducted with school officials who understood school policies and programmes related to the SSDU pillars. According to Wardhani et al. (2024), interviews will help researchers to understand better the obstacles that occur in implementing the SPAB programme. This study uses two variables, namely SSDU and GEDSI. The SSDU variable will focus on three indicators: Safer learning facilities, school safety and educational continuity management and risk reduction and resilience education (GADDRESS 2022). The GEDSI variable focuses on six indicators: School commitment, curriculum, information dissemination, school facilities, supervision system and cooperation.

### Data analysis

Data analysis in this study was conducted using two statistical approaches, namely descriptive analysis and regression analysis. Descriptive statistical analysis was used to determine the level of school readiness in implementing the SSDU based on GEDSI. Through this analysis, the data obtained from the questionnaire will be described in the form of a percentage to provide an overview of the readiness of each school based on the SSDU and GEDSI indicators. Furthermore, to test the influence of the level of implementation of GEDSI principles on the success of SSDU implementation in schools, a linear regression analysis technique was used. Regression analysis predicts significant dependencies between independent and dependent variables (Creswell & Creswell 2018). This analysis aims to determine the extent to which GEDSI principles contribute to the effectiveness of the SSDU programme implementation, as well as to identify key variables that play a role in increasing school resilience to disasters in an inclusive manner.

### Ethical considerations

Ethical clearance to conduct this study was obtained from the Universitas Muhammadiyah Surakarta, Health Research Ethics Committee (No. 1012/KEPK-FIK/VI/2025).

## Results

This section presents the results of the analysis on school preparedness for disaster resilience by examining two interrelated aspects: The readiness of schools to implement the SSDU and the integration of GEDSI principles within the Safe Schools Programme. The analysis not only describes the level of preparedness across key dimensions for each component but also explores the relationship between institutional disaster management readiness and the extent to which GEDSI is systematically embedded in school policies, systems and practices. By analysing these dimensions concurrently, this section provides empirical insights into whether stronger disaster preparedness structures are associated with more inclusive and equitable implementation of school-based DRR initiatives.

### Readiness of implementation Safe School Disaster Unit

An analysis of the three pillars of SSDU revealed significant variations in preparedness levels across schools in Sukoharjo Regency. This finding is important considering the region’s geographical location in a flood-prone area, where differences in capacity between schools can impact the effectiveness of the overall preparedness system.

The highest achievement in Pillar 1 was achieved by two schools: Public Senior High School 3 Sukoharjo (28.9%) and Private Senior High School 1 Sukoharjo (28.82%), far exceeding the achievements of other schools (Table 1). Conversely, the private school, Private Senior High School 5 Gatak, scored the lowest at 4.15%, indicating limitations in meeting standards for safe school physical facilities, such as risk-resistant building structures and environmental protection systems. The high disparity between schools indicates a gap in investment and management of physical facilities, which requires attention from the local government, given that facilities are a key foundation for reducing the risk of loss of life in disasters.

**TABLE 1:** Readiness of implementation of the Safe School Disaster Unit.

Descriptive statistics	Average	%
**Pillar 1. Safer school facilities**		
Public Senior High School 1 Mojolaban	29.0	12.04
Private Senior High School 5 Gatak	10.0	4.15
Public Senior High School 1 Nguter	62.8	26.08
Private Senior High School 1 Sukoharjo	69.4	28.82
Public Senior High School 3 Sukoharjo	69.6	28.90
**Pillar 2. Disaster management and educational continuity**		
Public Senior High School 1 Mojolaban	100.6	18.85
Private Senior High School 5 Gatak	20.2	3.78
Public Senior High School 1 Nguter	136.4	25.55
Private Senior High School 1 Sukoharjo	139.4	26.11
Public Senior High School 3 Sukoharjo	137.2	25.70
**Pillar 3. Risk reduction and resilience education**		
Public Senior High School 1 Mojolaban	53.3	14.13
Private Senior High School 5 Gatak	14.8	3.92
Public Senior High School 1 Nguter	81.0	21.47
Private Senior High School 1 Sukoharjo	121.8	32.29
Public Senior High School 3 Sukoharjo	106.3	28.18

Pillar 2 had the most equitable distribution of achievement compared to the other pillars. Three schools: Public Senior High School 1 Nguter (25.55%), Private Senior High School 1 Sukoharjo (26.11%) and Public Senior High School 3 Sukoharjo (25.70%) had relatively stable scores. This pattern indicates that disaster management and learning continuity mechanisms, such as evacuation standard operating procedures (SOPs), contingency planning and simulations, have been implemented more systematically in these schools. However, Private Senior High School 5 Gatak again showed the lowest score (3.78%), confirming structural issues in preparedness capacity, both in terms of resources and internal school policies.

Pillar 3 exhibits the most contrasting pattern. Private Senior High School 1 Sukoharjo achieved the highest achievement (32.29%), reflecting the strong integration of disaster education programmes, for example, through the curriculum, extracurricular activities and student engagement. On the other hand, Private Senior High School 5 Gatak again ranked lowest (3.92%), indicating a lack of active learning-based disaster education programmes or strengthening risk literacy. The differences between schools in Pillar 3 emphasise that disaster education is heavily influenced by internal policies, teacher support and partnerships with the Regional Disaster Management Agency (BPBD), humanitarian agencies and local communities.

The high achievement in Pillars 2 and 3 in several schools indicates that disaster management and education capacity are relatively more developed than physical facilities. This aligns with the national trend showing that schools adopt training and curriculum more quickly than infrastructure investments. This is because Pillars 2 and 3 are operationally low cost, can be implemented gradually and rely on human participation, rather than large physical investments. Because of their intangible nature, these managerial and educational aspects tend to grow more rapidly despite inadequate infrastructure. The analysis shows that the disparity in capacity between schools reflects not only administrative differences but also a form of non-physical vulnerability that can exacerbate disaster risk. Schools that consistently record low scores have the potential to become weak points in the region’s preparedness system.

### Readiness of implementation Gender, Disability, and Social Inclusion

The descriptive analysis reveals that the level of readiness for implementing GEDSI within the SSDU varies considerably across dimensions and between schools, particularly when comparing public and private institutions. In terms of school commitment, public senior high schools demonstrate a consistently higher level of readiness than private schools, with Public Senior High School 3 Sukoharjo recording the highest proportion (29.37%) (Table 2). This finding suggests that institutional leadership, internal policy support and alignment with government-driven inclusive education agendas play a crucial role in strengthening commitment to GEDSI, factors that are generally more pronounced in public schools.

**TABLE 2:** Readiness to implement Gender, Disability, and Social Inclusion.

Descriptive statistics	Average	%
**School commitment**		
Public Senior High School 1 Mojolaban	57.0	14.61
Private Senior High School 5 Gatak	14.6	3.74
Public Senior High School 1 Nguter	87.0	22.30
Private Senior High School 1 Sukoharjo	117.0	29.98
Public Senior High School 3 Sukoharjo	114.6	29.37
**School curriculum**		
Public Senior High School 1 Mojolaban	78.2	16.48
Private Senior High School 5 Gatak	17.4	3.67
Public Senior High School 1 Nguter	110.6	23.30
Private Senior High School 1 Sukoharjo	126.8	26.72
Public Senior High School 3 Sukoharjo	141.6	29.84
**Information dissemination**		
Public Senior High School 1 Mojolaban	94.0	17.00
Private Senior High School 5 Gatak	20.0	3.62
Public Senior High School 1 Nguter	135.0	24.41
Private Senior High School 1 Sukoharjo	135.0	24.41
Public Senior High School 3 Sukoharjo	169.0	30.56
**School facilities**		
Public Senior High School 1 Mojolaban	27.5	12.39
Private Senior High School 5 Gatak	9.5	4.28
Public Senior High School 1 Nguter	53.5	24.10
Private Senior High School 1 Sukoharjo	53.5	24.10
Public Senior High School 3 Sukoharjo	78.0	35.14
**Supervision system**		
Public Senior High School 1 Mojolaban	37.0	12.11
Private Senior High School 5 Gatak	15.0	4.91
Public Senior High School 1 Nguter	78.5	25.70
Private Senior High School 1 Sukoharjo	101.0	33.06
Public Senior High School 3 Sukoharjo	74.0	24.22
**Cooperation**		
Public Senior High School 1 Mojolaban	30.0	9.12
Private Senior High School 5 Gatak	10.5	3.19
Public Senior High School 1 Nguter	79.5	24.16
Private Senior High School 1 Sukoharjo	100.5	30.55
Public Senior High School 3 Sukoharjo	108.5	32.98

A similar pattern is observed in the school curriculum dimension, where Public Senior High School 3 Sukoharjo achieves the highest score (29.84%). This indicates that the integration of GEDSI principles into curricular content, teaching strategies and extracurricular activities is more advanced in certain public schools. In contrast, the relatively lower and uneven scores among private schools highlight gaps in systematically mainstreaming GEDSI into educational practices, pointing to the need for targeted capacity building and technical guidance.

The information dissemination dimension shows greater variation across schools. Although public schools still dominate the highest scores, particularly Public Senior High School 3 Sukoharjo (30.46%), several private schools demonstrate comparatively competitive performance. This suggests that inclusive disaster-related information dissemination is not solely dependent on institutional status but can also be influenced by school-level initiatives, communication practices and engagement with local communities.

With regard to the school system dimension, readiness for GEDSI implementation remains largely concentrated in public schools, with Public Senior High School 3 Sukoharjo achieving the highest percentage (35.14%). This dimension reflects structural and procedural preparedness, including planning mechanisms, role allocation and emergency response systems that are sensitive to the needs of vulnerable groups. The lower performance among private schools indicates that systemic and institutionalised approaches to GEDSI remain a significant challenge, particularly in ensuring sustainability.

The cooperation dimension also highlights the stronger position of public schools, with Public Senior High School 3 Sukoharjo attaining the highest score (32.98%). This underscores the importance of collaboration with external stakeholders such as local governments, civil society organisations and community groups in reinforcing GEDSI implementation. Overall, these findings indicate that readiness for integrating GEDSI into the SSDU Programme is uneven, with certain public schools acting as frontrunners, while private schools require more strategic and structured support to enable comprehensive and sustainable inclusive disaster resilience practices.

### Statistical assessment of Gender, Disability, and Social Inclusion implementation in the Safe School Disaster Unit

The results of the simple linear regression analysis in this study indicate that the integration of GEDSI principles has a positive and significant effect on the success of the SSDU Programme implementation in schools. Based on the output, a constant value of 8.101 and a regression coefficient for the GEDSI variable of 0.917 were obtained. This means that every one-unit increase in the GEDSI score will increase the SSDU implementation score by 0.917, assuming that other variables remain constant. The significance value (Sig.) obtained for the constant and regression coefficient is less than 0.001 (Sig. < 0.001), which indicates that this effect is statistically significant at a 99% confidence level (α = 0.01).

These results indicate that the higher the level of integration of GEDSI principles in schools, including gender equality, fulfilment of rights and access for people with disabilities and social inclusion in disaster policies and practices, the higher the level of success of the SSDU programme implementation. Schools that pay attention to inclusive aspects in simulation activities, emergency response plan preparation and involvement of the entire school community show more optimal preparedness. The SSDU programme basically aims to create a safe learning environment for the entire school community, and this goal will not be achieved optimally without considering the diversity of needs, roles and vulnerabilities of individuals in the school community. Thus, it can be concluded that GEDSI is an important factor that supports the effectiveness of SSDU implementation, so mainstreaming GEDSI principles needs to be an integral part of DRR strategies in educational environments. Without implementing GEDSI principles, the SSDU programme risks ignoring vulnerable groups and potentially creating inequality in preparedness efforts. Therefore, the results of this study emphasise the importance of GEDSI integration as a primary strategy to strengthen school resilience to disasters in a fair, equal and sustainable manner.

## Discussion

In more detail, the results show that in terms of infrastructure, most schools are not equipped with evacuation routes that are friendly for students with disabilities. Other supporting facilities, such as emergency shelters, must be adapted to meet the needs of groups with limited mobility. Many schools are built without considering disaster risks, especially in areas prone to earthquakes and floods. This is consistent with Widowati et al.’s (2023) research, which found that many schools are still located in disaster-prone areas. This increases the vulnerability of students and teachers and reduces the effectiveness of disaster mitigation programmes. The retrofitting process (strengthening buildings) also requires a lot of money and time.

These findings indicate that school readiness in implementing GEDSI and the SSDU programme still needs to be improved; it can even be said that no concrete actions have been taken to prepare schools to be more inclusive and responsive to disasters. This unpreparedness not only shows a need for physical facilities but also in terms of the capacity and awareness of school management regarding the importance of the role of GEDSI in DRR. One of the main factors causing low school readiness in implementing GEDSI is the lack of understanding of gender and disability vulnerabilities in the school environment. Many schools do not yet understand that vulnerable groups, such as women and people with disabilities, require special attention and treatment in disaster situations. This low level of understanding has resulted in the absence of policies and procedures that support the integration of GEDSI into school disaster programmes. Awareness of disaster risks and the steps that need to be taken to deal with emergencies is often limited to a theoretical level, without adequate practical application.

The low allocation of resources to facilitate the implementation of SSDU and GEDSI is also a major challenge. Schools that were the object of the study revealed that budget constraints and minimal support from local governments were obstacles to implementing safer and more inclusive infrastructure (Marlina, Ruslanjari & Hakim 2024). These limitations include physical facilities, training for educators and providing educational materials related to inclusive disaster mitigation. Many schools, especially in remote areas, need more infrastructure, expertise and funds to implement SSDU programmes effectively. The lack of budget makes it difficult for schools to update physical facilities to be disaster resistant and provide regular training for teachers and students on disaster preparedness (Ronggowulan et al. 2024).

The regulatory aspect is also an important highlight in this discussion. Although the government has initiated the SSDU and GEDSI programmes, their implementation at the school level is still hampered by the lack of binding and firm regulations, so they are not optimal. Regulations related to school disaster preparedness are only sometimes followed by clear implementation guidelines or adequate technical support (Fandayati et al. 2024). Many schools do not yet have policies that regulate sustainable resource planning. The absence of regulations requiring schools to include GEDSI components in their SSDU programmes has resulted in many schools not integrating these aspects into their disaster policies. As a result, no proactive steps have been taken to ensure that schools are safe for all groups, including women and students with disabilities. Worse still, many schools need to understand the importance of implementing SSDU because they consider their schools to be in safe areas.

From a policy perspective, these results highlight the need for further interventions from both government and educational institutions to promote the integration of GEDSI and SSDU across schools. Existing evacuation policies and procedures are still general and do not take into account the specific needs of female students and students with disabilities, such as protection from the risk of gender-based violence in disaster situations. Strengthening school capacity through specific training or improving facilities that support disaster preparedness should be a top priority. In addition, collaboration between schools, communities and other stakeholders is needed to create a safer and more inclusive education ecosystem for disasters. Schools’ efforts to involve local communities, parents and other stakeholders in the SSDU programme still need to be more optimal. Active participation from the community is essential to support the sustainability of this programme, but participatory approaches are still limited to a few schools. Community participation aims to maintain the implementation of the programme on an ongoing basis and allow for control over existing activities.

## Conclusion

This study concludes that school readiness in implementing the SSDU and integrating GEDSI principles remains uneven and generally limited across the sampled schools. Although schools demonstrate a foundational awareness of inclusive disaster preparedness, the quantitative evidence indicates that GEDSI has not yet been systematically embedded in institutional policies, infrastructure and operational practices. Readiness is highest in information dissemination, reflecting initial efforts to promote inclusive awareness, but remains weak in systemic and infrastructural dimensions that are critical for effective and equitable disaster response. The findings of this study reveal that limited accessibility of physical facilities and insufficient institutional mechanisms constitute the primary barriers to inclusive SSDU implementation. These constraints suggest that awareness alone is insufficient without parallel investments in inclusive infrastructure and organisational capacity. Importantly, the positive association between SSDU readiness and GEDSI integration identified in this study indicates that strengthening disaster management systems within schools can serve as a key entry point for advancing inclusive practices.

From a broader policy perspective, these results directly align with the objectives of the Sendai Framework for DRR (2015–2030), particularly Priority 4, which emphasises strengthening disaster preparedness for effective response while addressing the needs of vulnerable populations. The study also contributes to the achievement of the SDGs, especially SDG 4 (Quality Education) by promoting safe and inclusive learning environments, SDG 5 (Gender Equality), SDG 10 (Reduced Inequalities) and SDG 11 (Sustainable Cities and Communities) through inclusive DRR at the school level. Overall, the findings underscore the need for a shift from awareness-based interventions towards the institutionalisation of GEDSI within SSDU implementation. Strengthening policy commitment, investing in inclusive infrastructure and enhancing the capacity of school personnel are essential to advancing equitable school-based disaster resilience. By aligning SSDU programmes with the principles of the Sendai Framework and the SDGs, schools can play a strategic role in ensuring that DRR efforts leave no one behind.
